# ADD-Net: An Adaptive Discriminative Network for Multiclass Lesion Detection in Hysteroscopic Images

**DOI:** 10.3390/jimaging12100464

**Published:** 2026-09-22

**Authors:** Youfang Lu, Shunxiang Shi, Lidong Huang

**Affiliations:** College of Mathematics and Computer Science, Yunnan Minzu University, Kunming 650500, China

**Keywords:** hysteroscopic imaging, lesion detection, adaptive feature learning, prototype learning

## Abstract

Hysteroscopic lesion detection aims to automatically localize and classify lesions in hysteroscopic images, thereby facilitating intrauterine disease screening and clinical diagnosis. However, pronounced background clutter and interclass visual similarity in hysteroscopic images can impair feature discriminability. Although prevailing methods incorporate multiscale feature aggregation or local detail refinement, these operations rely on fixed processing schemes that constrain adaptive feature recalibration under varying imaging conditions, often resulting in representational instability and interclass confusion. To address this issue, we propose ADD-Net, an adaptive discriminative detection network tailored to multiclass hysteroscopic lesion detection. Specifically, a hybrid adaptive feature modulation strategy leverages an expert selection mechanism to dynamically recalibrate backbone features, improving model robustness under challenging hysteroscopic imaging conditions. A prototype confusion interaction mechanism enhances class discriminability by modeling class prototypes and interclass confusion relationships. In addition, the model uses lesion presence probabilities to improve detection stability. On the HS-CMU dataset, ADD-Net increases the three-run mean mAP@50 from 92.5% for Mamba YOLO-T to 93.3%, while reducing parameter count, computational cost, and inference latency by approximately 16.0%, 13.5%, and 33.3%, respectively. Experimental results demonstrate that ADD-Net improves detection performance while maintaining high computational efficiency, highlighting its potential for computer-aided diagnosis.

## 1. Introduction

Hysteroscopy is an essential tool for disease screening, lesion localization, and clinical diagnosis [1]. Automatic detection of lesions can improve screening efficiency and localization accuracy. However, reliable class discrimination remains challenging in hysteroscopic images. Fluid interference, tissue occlusion, and nonuniform illumination during hysteroscopic imaging obscure the boundaries between lesions and surrounding normal tissue. Moreover, different types of intrauterine lesions often exhibit substantial similarities in color, texture, and morphology. Together, these factors hinder the learning of robust and discriminative lesion representations, increasing the risk of interclass confusion. Therefore, learning sufficiently discriminative representations across lesion classes under challenging hysteroscopic imaging conditions remains a key challenge in improving detection performance.

Early approaches to hysteroscopic image analysis relied primarily on handcrafted features and conventional machine learning algorithms. These methods characterized lesions using color [2], texture [3], and edge descriptors [4], and combined these representations with support vector machines [5] or *k*-nearest-neighbor classifiers [6] for lesion recognition. Although these approaches have advanced automated hysteroscopic image analysis, they rely heavily on handcrafted feature engineering and are sensitive to image quality. Under substantial background clutter, handcrafted descriptors may fail to represent lesion regions reliably, thereby limiting the generalizability and clinical applicability of these methods.

With the development of deep learning, convolutional neural networks (CNNs) have been adopted for automated hysteroscopic image analysis [7,8,9,10,11]. Through hierarchical feature extraction, CNNs can automatically capture local textures, edge structures, and high-level semantic representations, showing favorable performance in medical image classification, segmentation, and object detection. Building on these advances, single-stage object detectors exemplified by the You Only Look Once (YOLO) family have further improved lesion localization efficiency [12,13,14,15]. Recent studies have pursued targeted improvements in hysteroscopic lesion detection, including lightweight architectures [16], multiscale feature fusion [17], and the enhancement of boundary details [18]. Nevertheless, most existing approaches rely on generic feature enhancement strategies and remain limited in learning discriminative representations across lesion classes under challenging hysteroscopic imaging conditions. Specifically, (1) hysteroscopic images are frequently affected by complex imaging artifacts. Conventional backbone networks struggle to simultaneously model fine-grained textures, structural context, and illumination variations, resulting in unstable lesion representations. (2) Different categories of hysteroscopic lesions exhibit substantial visual similarity. Learning class decision boundaries directly from fused features, without explicitly modeling class-level semantic priors and relationships among confusable categories, can therefore lead to frequent misclassification.

To address these limitations, we propose ADD-Net, an adaptive discriminative network for multiclass hysteroscopic lesion detection. As illustrated in Figure 1, ADD-Net differs from existing approaches by jointly modeling feature adaptation and class discrimination, thereby improving discriminative representations of lesions under challenging hysteroscopic imaging conditions. First, we design the Hybrid Adaptive Feature Modulation (HAFM) module. Its local-texture, structural-context, and illumination-robustness experts model fine-grained textures, irregular structures, and illumination artifacts, respectively. A sparse gating mechanism dynamically selects the most relevant expert outputs, enabling backbone features to better adapt to challenging hysteroscopic imaging conditions. Second, we introduce the Prototype Confusion Interaction Module (PCIM), which injects class-semantic priors into the fused neck features and sharpens the decision boundaries among visually similar lesion categories. Finally, the Lesion Presence Calibration Head (LPC-Head) introduces a class-agnostic lesion presence branch that explicitly models lesion responses at candidate locations, further improving detection robustness.

The main contributions of this work are summarized as follows:

(1) We propose ADD-Net, an adaptive discriminative network for multiclass hysteroscopic lesion detection. ADD-Net jointly improves feature adaptation and class discrimination to address the limited discriminability of lesion representations under challenging hysteroscopic imaging conditions.
(2) We develop HAFM to improve the model’s adaptability to complex textures, irregular structures, and illumination variations in hysteroscopic images, thereby producing more stable lesion representations.
(3) We introduce PCIM, which incorporates class prototype priors and relationships among confusable categories into the fused neck features. This design improves feature discriminability and reduces misclassification among visually similar lesion categories.
(4) ADD-Net achieves a three-run mean mAP@50 of 93.3% on the HS-CMU dataset, outperforming the baseline by 0.8 percentage points, while reducing parameter count, computational cost, and inference latency by approximately 16.0%, 13.5%, and 33.3%, respectively. These results demonstrate its effectiveness in multiclass hysteroscopic lesion detection.

## 2. Related Work

### 2.1. Deep Learning-Based Hysteroscopic Lesion Detection

Quantitative feature extraction is a fundamental component of medical image analysis. Before the widespread adoption of deep learning, related methods relied primarily on manually designed quantitative imaging features and imaging biomarkers. Radiomics further systematized this analytical paradigm by extracting predefined intensity, shape, and texture features from regions of interest to quantitatively characterize tissue phenotypes. Although these features generally offer good interpretability, their stability and reproducibility can be affected by variations in image acquisition, preprocessing, region-of-interest segmentation, and feature computation pipelines [19]. More recently, deep learning has enabled models to learn hierarchical, task-specific features directly from images, promoting a gradual shift in medical image analysis from handcrafted feature engineering toward data-driven representation learning. In hysteroscopic image analysis, methods based on learned representations can be broadly divided into two categories. The first comprises image-level diagnostic and classification methods [20,21], which primarily focus on benign–malignant discrimination, lesion-type recognition, and severity grading. The second includes object detection methods for lesion localization [16,17,18], which jointly predict lesion locations and categories and are therefore better aligned with clinical needs for lesion screening, localization, and diagnostic support.

For image-level diagnosis and classification, Zhang et al. developed a VGG-16-based model to automatically classify multiple types of endometrial lesions in hysteroscopic images. Their results demonstrated the effectiveness of CNNs for computer-aided diagnosis in hysteroscopy [20]. Raimondo et al. proposed a deep learning model for identifying and classifying intrauterine lesions in hysteroscopic images. They further investigated the effects of clinical factors on diagnostic performance [21]. These methods effectively learn diagnostically relevant image-level representations and support computer-aided diagnosis, but their inability to directly localize lesions limits their utility for clinical lesion localization.

Compared with image-level classification, object detection provides explicit lesion-level spatial information, making it better suited for hysteroscopic lesion localization and real-time diagnostic support during hysteroscopy. Chen et al. proposed HD-YOLO, a lightweight YOLO11-based detector that enhances feature representations of benign and malignant endometrial lesions while reducing model complexity and maintaining real-time performance [16]. Zhang and Peng proposed YOLOv11-LC, which integrates the Laplacian coordinate attention and the CMA-C3k2 module into YOLOv11 to mitigate performance degradation caused by nonuniform illumination, lesion-scale variation, and texture interference in hysteroscopic images [17]. Furthermore, Chen et al. proposed HL-Det, a lightweight single-stage detector that combines dual-domain feature modeling with dynamic sampling to enhance the representation of lesion boundaries and fine-grained structures across multiple scales, thereby improving lesion localization in challenging hysteroscopic images [18]. These approaches have advanced hysteroscopic lesion localization and real-time detection, particularly through lightweight architectures, multiscale feature fusion, and boundary detail enhancement. Nevertheless, most emphasize generic feature enhancement and provide limited mechanisms for adapting representations to challenging imaging conditions or explicitly modeling confusion among visually similar lesion categories.

### 2.2. Adaptive Feature Learning

High-quality annotated data are often scarce in medical image analysis, and therefore many deep learning models adopt feature extractors pretrained on ImageNet or medical imaging datasets. These models are then fine-tuned to transfer general visual representations to specific medical imaging tasks. Taher et al. conducted a large-scale evaluation of the transferability of multiple pretrained models across medical imaging tasks. Their results showed that pretraining provides effective parameter initialization and strong transfer potential across a range of downstream tasks [22]. Valverde et al. systematically analyzed factors affecting the performance of ImageNet-pretrained models in medical image classification, providing guidance for model selection and fine-tuning in medical imaging applications [23]. Okazaki et al. compared the transfer performance of models pretrained on RadImageNet and ImageNet across medical imaging tasks, demonstrating the value of domain-specific pretraining in specialized medical applications [24]. These studies indicate that appropriately selected and fine-tuned pretrained models provide robust initial representations for medical imaging tasks, improving training stability and generalization.

Although pretrained weights provide general edge, texture, and structural representations that improve training stability and reduce the risk of overfitting, substantial differences remain between natural and medical images. Directly using pretrained features may therefore be insufficient for adapting to variations in lesion characteristics in complex medical images. To improve model adaptability to complex medical image features, recent studies have explored adaptive feature processing and dynamic feature fusion. Li et al. proposed EAFP-Med, an adaptive feature processing module that leverages prompt-driven dynamic parameter updates to extract cross-domain and multiscale lesion features from diverse medical images, improving complex lesion representation in medical image detectors [25]. Cai et al. proposed DAFNet, a dynamic adaptive fusion network that combines dynamic feature fusion with attention mechanisms to strengthen salient feature representations and improve adaptation to feature variability in complex medical images [26]. These studies indicate that adaptive feature processing enables medical imaging models to better accommodate heterogeneous input distributions, providing a viable strategy for hysteroscopic lesion detection.

### 2.3. Prototype-Guided Discriminative Learning

Prototype learning enhances feature discriminability through class-level semantic representations and has recently gained broad adoption in medical image analysis. Zhang et al. proposed Prototype Correlation Matching and Class-Relation Reasoning (PMCR), which addresses substantial intraclass variation and inadequate modeling of interclass relationships through prototype matching and class-relation reasoning [27]. Song et al. introduced a network based on interactive prototype learning and self-learning that strengthens intraclass consistency while reducing interclass similarity through prototype interactions [28]. Wang et al. further developed a cross- and intra-image prototypical learning framework for multi-label disease diagnosis and interpretation, improving prototype representations under complex pathological contexts [29]. Collectively, these studies demonstrate that prototype learning and class-relation modeling can enhance discriminative representations in medical image analysis. However, existing methods primarily focus on medical image classification or segmentation, leaving confusion among visually similar categories in multiclass hysteroscopic lesion detection largely underexplored. Moreover, conventional prototype interaction mechanisms typically emphasize matching local features with class prototypes, while providing only limited support for modeling global discriminative interactions among class-relevant regions.

## 3. Materials and Methods

### 3.1. Datasets

#### 3.1.1. HS-CMU Dataset

We evaluated the proposed method on the HS-CMU hysteroscopic lesion dataset provided by the Department of Obstetrics and Gynecology at Beijing Chaoyang Hospital, Capital Medical University [30]. Representative images are shown in Figure 2a. The original dataset contains 3385 lesion images from eight categories: submucosal myoma (SM), endometrial cancer (EC), endometrial polyp (EP), endometrial polypoid hyperplasia (EPH), endometrial hyperplasia without atypia (EH), intrauterine foreign body (IFB), cervical polyp (CP), and atypical endometrial hyperplasia (AHE).

Before model training, all images underwent targeted quality control under the supervision of two clinicians with master’s degrees. During this procedure, 20 images containing only isolated point annotations or depicting lesions whose categories could not be reliably determined were excluded, and inaccurate bounding boxes in 84 images were corrected in accordance with the original annotation protocol. Conflicting category labels in 179 images were reassessed using the same protocol, with unresolved cases adjudicated by a chief physician specializing in gynecologic endoscopy. After filtering and correction, 3365 valid images remained. To prevent data leakage, the dataset was partitioned using a case-level stratified group split. The substring preceding the final underscore in each filename was treated as the case identifier, and all images sharing the same identifier were assigned exclusively to one subset. The resulting training, validation, and test sets contained 2682, 349, and 334 images, respectively, corresponding to an approximate ratio of 8:1:1. Additionally, to facilitate the assessment of class balance, Table 1 reports the number of annotated lesion instances in each category for the training, validation, and test sets. Because an individual image may contain multiple annotated lesions, the values in Table 1 represent lesion instances rather than images.

#### 3.1.2. Kvasir-Capsule Dataset

To assess the cross-dataset applicability of the proposed architecture, we conducted an independent experiment on the Kvasir-Capsule dataset [31]. The original dataset contains approximately 4.74 million frames collected from 117 wireless capsule endoscopy examinations. Among these frames, 47,238 were annotated by experts and grouped into 14 gastrointestinal categories.

Because the present experiment focused on multiclass localization of clinically relevant abnormal findings, we retained eight target categories: erosion (ERO), angiectasia (ANG), erythema (ERY), fresh blood (FBL), foreign body (FOB), ulcer (ULC), polyp (POL), and lymphangiectasia (LYM). The ampulla of Vater, ileocecal valve, and pylorus were excluded because they represent anatomical landmarks rather than lesions, whereas normal clean mucosa and reduced mucosal view represent normal or limited-visibility observations rather than target abnormalities. Blood–hematin was also excluded because its extremely small number of annotated images was insufficient to support a reliable training, validation, and test split. Category filtering and data curation yielded a final dataset of 4212 images, with representative samples shown in Figure 2b. The dataset was partitioned using a video-level stratified group split, with all images originating from the same capsule endoscopy examination assigned exclusively to one subset. The resulting training, validation, and test sets contained 3365, 417, and 430 images, respectively, corresponding to an approximate ratio of 8:1:1.

### 3.2. Overall Architecture

Figure 3 illustrates the overall architecture of ADD-Net, which extends Mamba YOLO-T [32] to enhance feature adaptability and class discriminability for multiclass lesion detection in complex hysteroscopic images. EfficientNet-B0 [33] serves as the backbone feature extractor, with the Hybrid Adaptive Feature Modulation (HAFM) module dynamically recalibrating the backbone’s multiscale outputs to improve robustness to imaging interference. Subsequently, a Prototype Confusion Interaction Module (PCIM) is embedded in multiscale fusion to model class prototypes and interclass confusion relationships, enhancing fused-feature discriminability and reducing misclassification among visually similar hysteroscopic lesion categories. The Lesion Presence Calibration Head (LPC-Head) generates predictions from feature maps at three scales.

### 3.3. Hybrid Adaptive Feature Modulation

Hysteroscopic image analysis is often degraded by nonuniform illumination, tissue occlusion, and irregular lesion morphology. Backbones pretrained on natural images face a domain gap that limits their ability to jointly capture local textures and structural context while accommodating illumination variation, potentially resulting in unstable lesion representations. To address this limitation, we introduce HAFM, whose architecture is shown in Figure 3a. HAFM dynamically modulates backbone features using a set of complementary experts and a sparse gating mechanism, thereby improving the model’s adaptability to challenging hysteroscopic imaging conditions.

Given a feature map $F\in R^{C\times H\times W}$ produced by the pretrained backbone, HAFM first applies a 1×1 convolution followed by a SiLU activation to project the original feature into $F_{b}\in R^{C_{b}\times H\times W}$. This projection reduces channel redundancy and the computational cost of the subsequent expert branches. Based on $F_{b}$, HAFM constructs three complementary experts for local-texture enhancement, structural-context modeling, and illumination-robust modulation, respectively.

The Local Texture Expert (LTE) is designed to enhance local tissue textures and fine-grained lesion details in hysteroscopic images. The input feature is first processed by a 3×3 depthwise convolution to capture channel-wise texture variations and edge responses within local neighborhoods. A subsequent 1×1 pointwise convolution enables cross-channel interaction and aggregates complementary texture information from different channels.

(1) $$E_{1}={\mathrm{Conv}}_{1\times 1}\left({\mathrm{DWConv}}_{3\times 3}\left(F_{b}\right)\right).$$

The Structural Context Expert (SCE) models lesion morphology and the surrounding tissue context. It processes the input feature using a 3×3 dilated depthwise convolution with a dilation rate of d=2. This operation enlarges the effective receptive field while preserving spatial resolution and maintaining low computational cost, enabling the aggregation of contextual information from the lesion and its neighboring tissues.

(2) $$E_{2}={\mathrm{Conv}}_{1\times 1}\left({\mathrm{DWConv}}_{3\times 3}^{d=2}\left(F_{b}\right)\right).$$

The Illumination-Robustness Expert (IRE) is designed to handle common imaging artifacts in hysteroscopic images, including specular reflections, low contrast, and nonuniform illumination. The input feature is first processed by global average pooling to obtain a global statistical descriptor that captures the overall illumination distribution and channel responses. A subsequent 1×1 convolution generates channel-wise modulation weights for adaptive feature recalibration. Finally, a 3×3 depthwise convolution performs spatial refinement.

(3) $$\left\{\begin{array}{cc} w_{c} & =\sigma_{h}\left(\phi_{c}\left(\mathrm{GAP}(F_{b})\right)\right),\\ E_{3} & ={\mathrm{Conv}}_{1\times 1}\left({\mathrm{DWConv}}_{3\times 3}\left(F_{b}⊙w_{c}\right)\right), \end{array}\right.$$

where GAP(·) denotes global average pooling, $\phi_{c}\left(\cdot \right)$ represents a channel mapping function composed of a 1×1 convolution and a nonlinear activation, $\sigma_{h}\left(\cdot \right)$ denotes the HardSigmoid activation function, and ⊙ indicates channel-wise multiplication.

To select the most appropriate expert combination for each input, HAFM employs a top-*K* sparse gating mechanism. The gating network first applies global average pooling and global max pooling to the original input feature *F*, and concatenates the resulting descriptors to form a global statistical vector. A lightweight 1×1 convolution then maps this vector to the response scores of the three experts.

(4) $$\left\{\begin{array}{cc} z & =\left[\mathrm{GAP}(F),\mathrm{GMP}(F)\right],\\ s & =\phi_{g}\left(z\right), \end{array}\right.$$

where GMP(·) denotes global max pooling, $s=[s_{1},s_{2},s_{3}]$ contains the gating scores of the three experts, and $\phi_{g}\left(\cdot \right)$ represents the gating-mapping function.

For each input, HAFM retains the two experts with the highest scores (K=2) and masks out the remaining expert. The selected scores are then normalized using a temperature-scaled softmax with $\tau_{h}=2.0$ to obtain the fusion weights $g_{i}$. Because the weights of the unselected experts is zero, HAFM fuses only the two expert branches most relevant to the current input. The fused expert response is then projected back to the original channel dimension through a 1×1 channel-mapping layer and combined with the input feature through a residual connection to produce the final output.

(5) $$\left\{\begin{array}{cc} F_{\mathrm{moe}} & =\sum_{i=1}^{3}g_{i}E_{i},\\ \Delta F & =\phi_{p}\left(F_{\mathrm{moe}}\right),\\ F^{\prime } & =F+\alpha_{h}\Delta F, \end{array}\right.$$

where $\phi_{p}\left(\cdot \right)$ denotes the 1×1 channel projection operation, and $\alpha_{h}$ is a learnable residual-scaling coefficient.

### 3.4. Prototype Confusion Interaction Module

Although adaptive feature modulation improves feature adaptation to medical imagery, different categories of hysteroscopic lesions often remain visually similar in color, texture, and morphology. Learning class boundaries solely from fused features cannot fully exploit class-semantic priors and relationships among confusable categories, which can lead to misclassification. To address this limitation, we introduce PCIM, whose architecture is shown in Figure 3b. Unlike conventional convolution-based enhancement methods, PCIM explicitly constructs learnable class prototypes and injects class-semantic priors into spatial feature representations through interactions between image features and these prototypes. Given the substantial visual similarity among hysteroscopic lesion categories, PCIM further models inter-prototype similarities to generate class-confusion-aware prototypes. These refined prototypes strengthen the decision boundaries among visually similar categories.

To introduce class-level discriminative priors, PCIM maintains a set of learnable class prototypes. The prototype elements are independently initialized from a zero-mean Gaussian distribution with a standard deviation of 0.02, i.e., $N(0,0.02^{2})$. Each prototype represents the semantic center of its corresponding class in the latent feature space and is jointly optimized with the network parameters during training. During every forward pass, the class prototypes are further normalized to stabilize similarity computation.

(6) $$\left\{\begin{array}{cc} P & ={\left[p_{1},p_{2},\ldots ,p_{N_{c}}\right]}^{⊤}\in R^{N_{c}\times C_{h}},\\ {\hat{p}}_{c} & =\mathrm{Norm}\left(p_{c}\right),\;c=1,2,\ldots ,N_{c}, \end{array}\right.$$

Here, $N_{c}$ denotes the number of foreground target categories, and PCIM maintains one learnable prototype for each category. Therefore, the prototype set contains $N_{c}$ prototypes—eight for both the HS-CMU dataset and the selected Kvasir-Capsule categories in the present experiments. No background prototype is constructed because background regions do not constitute a single semantically coherent target category and are not included in the interclass prototype-relation modeling. Instead, candidate locations that are not assigned to any ground-truth target are handled as background by the detection-head supervision.

Given the substantial visual similarity among hysteroscopic lesion categories, PCIM further incorporates a class-confusion-aware prototype refinement mechanism. It first computes a pairwise similarity matrix among the normalized class prototypes. The diagonal elements are excluded to prevent each prototype from participating in its own confusion modeling. For the *c*-th prototype, the top-*k* most similar classes are selected to form its confusable-class index set $I_{c}$, where *k* was set to 2 in all experiments.

(7) $$\left\{\begin{array}{cc} S & =\hat{P}\;{\hat{P}}^{⊤},\\ I_{c} & =\mathrm{TopK}\left(S_{c},k\right), \end{array}\right.$$

where $S\in R^{N_{c}\times N_{c}}$ denotes the class-prototype similarity matrix.

The prototypes of the confusable classes are then aggregated using similarity-based weights.

(8) $$\left\{\begin{array}{cc} w_{cj} & =\frac{\exp(S_{cj}/\tau )}{\sum_{\ell \in I_{c}}\exp\left(S_{c\ell }/\tau \right)},\;j\in I_{c},\\ q_{c} & =\sum_{j\in I_{c}}w_{cj}{\hat{p}}_{j}, \end{array}\right.$$

where τ denotes the prototype temperature parameter, and $q_{c}$ represents the confusion context of the *c*th class.

Finally, each class prototype is residually fused with its confusion context to obtain a class-confusion-aware prototype.

(9) $${\tilde{p}}_{c}=\mathrm{Norm}\left({\hat{p}}_{c}+q_{c}\right).$$

PCIM further enables interactions between the confusion-aware class prototypes and pixel-level features. Specifically, the input feature $F^{\prime }\in R^{B\times C_{m}\times H\times W}$ is first projected along the channel dimension and flattened into a spatial sequence $F_{s}$. Before the pixel-to-prototype similarities are computed, both the projected pixel-feature vectors and the refined prototypes are normalized along the feature dimension to ensure numerical consistency. Similarity scores between each spatial position and the confusion-aware class prototypes are then computed, and a softmax function converts the resulting similarities into prototype response weights. The class prototypes are subsequently aggregated according to these weights to generate the prototype-guided context feature $F_{p}$. Finally, $F_{p}$ is reshaped into a spatial feature map $F_{p}^{\mathrm{map}}\in R^{B\times C_{h}\times H\times W}$. Through this interaction, each spatial position acquires class-semantic context according to its correspondence with different class prototypes, thereby improving the class discriminability of the fused features.

After the feature interaction, the original hidden feature $F^{\prime }$ is concatenated with the prototype-guided context feature $F_{p}^{\mathrm{map}}$. The concatenated features are integrated through a prototype-guided fusion operation comprising a 1×1 convolution, a 3×3 depthwise convolution, and another 1×1 convolution, yielding the fused feature $F_{g}$. Subsequently, the fused feature $F_{g}$ is fed into the SS2D [34] operator inherited from Mamba YOLO-T. Unlike the baseline, where SS2D directly models spatial dependencies in conventionally fused neck features, PCIM first injects confusion-aware class-prototype information into $F_{g}$ through pixel-to-prototype interaction and feature fusion. SS2D then models spatial dependencies within this prototype-conditioned representation, allowing class-discriminative information to propagate across lesion-relevant spatial locations and thereby improving the distinction between visually similar lesion categories. Finally, the output is projected back to the target channel dimension and residually added to the input feature to obtain the final output.

(10) $$\left\{\begin{array}{cc} F_{m} & =\;SS2D(F_{g}),\\ \Delta F^{\prime } & =\phi_{o}\left(F_{m}\right),\\ Y & =F^{\prime }+\alpha_{p}\Delta F^{\prime }. \end{array}\right.$$

### 3.5. Lesion Presence Calibration Head

HAFM and PCIM improve feature adaptation under challenging imaging conditions and strengthen discrimination among visually similar lesion categories, respectively. However, the classification branch of a conventional decoupled detection head must simultaneously perform lesion-category discrimination and background suppression. For lesion regions with weak class-specific cues, the classification response may remain susceptible to background interference. Consequently, the enhanced upstream features may not be fully translated into stable detection results. To further improve detection robustness in challenging hysteroscopic scenes, we develop LPC-Head based on the conventional decoupled detection head, as illustrated in Figure 3c. LPC-Head introduces a class-agnostic lesion presence branch that explicitly estimates whether each candidate location contains a lesion. The resulting lesion presence response is then used to calibrate the class predictions.

Given the input feature $F_{i}$ at the *i*-th detection scale, the regression and classification branches generate the bounding-box regression output and class prediction scores, respectively.

(11) $$\left\{\begin{array}{cc} B_{i} & =H_{\mathrm{reg}}^{i}\left(F_{i}\right),\\ C_{i} & =H_{\mathrm{cls}}^{i}\left(F_{i}\right), \end{array}\right.$$

where $B_{i}$ denotes the bounding box regression output, $C_{i}$ represents the class-prediction scores, and $H_{\mathrm{reg}}^{i}\left(\cdot \right)$ and $H_{\mathrm{cls}}^{i}\left(\cdot \right)$ denote the regression and classification branches at the *i*th detection scale, respectively.

Building on this decoupled structure, LPC-Head introduces a lightweight lesion presence branch that generates class-agnostic lesion responses. The branch produces a single-channel response map representing the likelihood that each spatial location contains a lesion. These lesion presence responses are then used to calibrate the class prediction scores.

(12) $$\left\{\begin{array}{cc} L_{i} & =H_{\mathrm{les}}^{i}\left(F_{i}\right),\\ {\tilde{C}}_{i} & =C_{i}+\alpha_{l}\;\tanh\left(L_{i}\right), \end{array}\right.$$

where $H_{\mathrm{les}}^{i}\left(\cdot \right)$ consists of a 1×1 convolution, a 3×3 depthwise convolution, and a final 1×1 convolution. The learnable scaling factor $\alpha_{l}$ controls the influence of the lesion presence response on the class-prediction scores.

Finally, the output at the *i*th detection scale is expressed as:

(13) $$Y_{i}=\mathrm{Concat}\left(B_{i},{\tilde{C}}_{i}\right).$$

During training, the lesion presence branch receives additional class-agnostic auxiliary supervision. Lesion presence labels are automatically constructed from the positive and negative sample locations determined by the task-aligned assigner. Locations assigned to ground-truth lesions are labeled as lesion-present, whereas all other locations are labeled as background. The lesion presence target is defined as:

(14) $$T_{j}^{\mathrm{les}}=\left\{\begin{array}{cc} 1, & j\in \Omega_{\mathrm{pos}},\\ 0, & j\notin \Omega_{\mathrm{pos}}, \end{array}\right.$$

where $\Omega_{\mathrm{pos}}$ denotes the set of positive locations determined by the task-aligned assigner, and *j* indexes the candidate locations.

The auxiliary lesion presence loss is computed using binary cross-entropy (BCE):

(15) $$L_{\mathrm{les}}=\frac{1}{\sum_{j}w_{j}}\sum_{j}w_{j}\;\mathrm{BCE}\left(L_{j},T_{j}^{\mathrm{les}}\right),$$

where $w_{j}$ denotes the loss weight assigned to the *j*th candidate location.

The final training objective is formulated as:

(16) $$L=\lambda_{\mathrm{box}}L_{\mathrm{box}}+\lambda_{\mathrm{cls}}L_{\mathrm{cls}}+\lambda_{\mathrm{dfl}}L_{\mathrm{dfl}}+\lambda_{\mathrm{les}}L_{\mathrm{les}},$$

where $L_{\mathrm{box}}$, $L_{\mathrm{cls}}$, and $L_{\mathrm{dfl}}$ denote the bounding-box regression loss, classification loss, and distribution focal loss, respectively. Moreover, $L_{\mathrm{les}}$ represents the auxiliary lesion presence loss, and the corresponding λ terms are loss-balancing coefficients.

### 3.6. Experimental Settings

All experiments were conducted on an NVIDIA GeForce RTX 4090 GPU with 24 GB of memory (NVIDIA Corporation, Santa Clara, CA, USA). The software environment comprised CUDA 12.4, Python 3.11.13, and PyTorch 2.1.2. We adopted a partial fine-tuning strategy for the ImageNet-pretrained EfficientNet-B0 backbone. Throughout training, only the final two feature extraction stages of the backbone were fine-tuned, while all preceding stages remained frozen. During training, the data augmentation settings followed the default configuration of the official Mamba YOLO-T implementation, except that the probability of Mosaic augmentation was set to 0.3. Mosaic augmentation was disabled during the final 20 training epochs. For the main comparison, ablation, and class-wise experiments, the random seed was set to 0. Two additional runs using random seeds of 21 and 42 were conducted for the repeated-run stability analysis. The remaining training hyperparameters are summarized in Table 2.

### 3.7. Evaluation Metrics

To comprehensively assess the proposed model, we use precision (P), recall (R), and mean average precision (mAP) as evaluation metrics. In addition, the number of model parameters (Params), floating-point operations (FLOPs), and inference latency are used to evaluate computational efficiency. The corresponding definitions are given below:

(17) $$\mathrm{Precision}=\frac{TP}{TP+FP},$$

(18) $$\mathrm{Recall}=\frac{TP}{TP+FN},$$

(19) $$\left\{\begin{array}{cc} {\mathrm{AP}}_{i} & =\int_{0}^{1}P_{i}\left(R\right)\;dR,\\ \mathrm{mAP} & =\frac{1}{N}\sum_{i=1}^{N}{\mathrm{AP}}_{i}, \end{array}\right.$$

Here, TP represents the number of samples for which the model correctly predicted the lesion type, FP represents the number of samples for which the model incorrectly predicted the lesion type, FN represents the number of samples missed by the model, and *N* denotes the total number of lesion categories.

## 4. Results

### 4.1. Backbone Comparison

In lesion detection models, the backbone extracts hierarchical visual representations whose quality directly affects the performance of the downstream neck and detection head. A suitable backbone should provide strong representational capacity while maintaining acceptable model complexity and inference efficiency. To select the base feature extractor for ADD-Net, we compared several representative backbone networks under identical experimental settings. The results are reported in Table 3.

As shown in Table 3, different backbone networks exhibit distinct trade-offs between detection accuracy and computational complexity. ResNet-50 achieves a relatively high mAP@50 of 92.5%, but requires 27.3 M parameters and 75.9 G FLOPs, resulting in substantial computational overhead. ConvNeXt-Tiny obtains an mAP@50 of 92.4%, while its parameter count and FLOPs increase to 31.2 M and 80.5 G, respectively. DenseNet-121 achieves an mAP@50:95 of 62.5%, but requires 54.9 G FLOPs and has an inference latency of 3.3 ms. These results indicate that deeper or more complex backbones can provide competitive detection performance, but often incur considerable computational costs.

Lightweight backbone networks can reduce model complexity, but often at the cost of detection accuracy. MobileNet-V2 contains only 5.7 M parameters and requires 12.8 G FLOPs. However, its mAP@50 and mAP@50:95 are 91.7% and 61.1%, respectively, both lower than those of EfficientNet-B0. RegNetY-800MF achieves an mAP@50 of 92.0% and an mAP@50:95 of 62.4%, which also remain below the corresponding results of EfficientNet-B0. Moreover, RegNetY-800MF requires 22.2 G FLOPs, compared with 14.8 G for EfficientNet-B0. These results suggest that simply adopting a smaller backbone may weaken the representation of lesion characteristics, particularly in hysteroscopic images containing subtle texture differences, irregular lesion boundaries, and substantial illumination artifacts.

EfficientNet-B0 offers a favorable accuracy–complexity trade-off, maintaining reliable detection performance without excessive model size or computational overhead. Therefore, we select EfficientNet-B0 as the backbone of ADD-Net.

### 4.2. Ablation Studies

#### 4.2.1. Overall Component Ablation Based on Mamba YOLO-T

Table 4 reports systematic ablation experiments conducted on the HS-CMU dataset to evaluate each module, with A, B, and C denoting HAFM, PCIM, and LPC-Head, respectively.

HAFM increases recall from 86.7% to 90.9%, confirming its effectiveness in adapting features to weak-boundary and low-contrast lesions. PCIM improves mAP@50 from 92.6% to 93.1%, while reducing the parameter count from 7.5 M to 6.1 M and inference latency from 2.7 ms to 1.8 ms. When used alone, LPC-Head slightly improves precision and recall but decreases mAP@50 from 92.6% to 92.3%. This indicates that its primary role is to calibrate class predictions through class-agnostic lesion presence modeling rather than independently enhance feature representations. When added to HAFM and PCIM, however, it increases recall from 88.7% to 89.6% and mAP@50 from 92.9% to 93.5%. The complete model achieves 93.3% precision, 89.6% recall, and the highest mAP@50 of 93.5%, with only 6.3 M parameters and 1.8 ms latency. Compared with the baseline, it improves recall and mAP@50 by 2.9 and 0.9 percentage points, while reducing the parameter count and latency by 16.0% and 33.3%, respectively. These results support the complementary contributions of feature adaptation, class discrimination, and lesion presence-aware calibration.

#### 4.2.2. Ablation of HAFM Expert Branches Within ADD-Net

To assess the contribution of each expert branch within HAFM, we compared the complete module with each of its three constituent branches, with the results reported in Table 5.

The three experts exhibit distinct functional contributions. Removing LTE reduces mAP@50 and mAP@50:95 to 92.8% and 62.1%, respectively, indicating that local-texture modeling improves the representation of fine-grained tissue patterns and lesions with weak boundaries. Removing SCE decreases recall to 84.5% and mAP@50 to 92.0%, representing the most pronounced degradation among the single-expert ablations. This result highlights the importance of structural context for detecting lesions with irregular morphology, poorly defined boundaries, or strong dependence on surrounding tissue. Removing IRE reduces precision from 93.3% to 90.2%, demonstrating that illumination-robust modulation mitigates the effects of local overexposure and nonuniform illumination, thereby improving prediction reliability.

The complete HAFM achieves the best results on multiple evaluation metrics. Notably, removing any individual expert produces only marginal changes in the parameter count, yet consistently degrades detection performance to varying degrees. This finding indicates that the improvement achieved by the complete module does not arise merely from increased network capacity, but primarily from the complementary contributions of the three experts.

#### 4.2.3. Ablation Study on PCIM Placement Within ADD-Net

To investigate the effect of PCIM placement, we adopted a progressive replacement strategy in which the XSSBlock modules of the original Mamba YOLO-T network were successively replaced with PCIM. The results are reported in Table 6. Throughout this placement study, HAFM and LPC-Head were kept enabled, and only the placement of PCIM was varied. Applying PCIM at all designated feature interaction stages corresponds to the complete ADD-Net configuration.

The benefits of PCIM for multiscale feature modeling become evident only when it is deployed across a sufficient number of feature-interaction stages. Replacing only the first XSSBlock decreases mAP@50 from 92.7% to 92.5%. When the first two XSSBlock modules are replaced, mAP@50 further decreases to 92.1%. These results indicate that introducing PCIM at only a few stages is insufficient to establish consistent class-discriminative representations across multiple scales. Moreover, combining PCIM with the original interaction modules does not fully exploit the advantages of PCIM.

When PCIM is applied to the first three feature-interaction stages, both detection performance and computational efficiency improve. The model achieves a recall of 88.2% and an mAP@50 of 93.0%, while reducing the parameter count, FLOPs, and inference latency to 7.1 M, 13.4 G, and 1.9 ms, respectively. Full replacement increases recall and mAP@50 by 2.6 and 0.8 percentage points, respectively, compared with the model without PCIM. It also reduces the parameter count, FLOPs, and inference latency by approximately 17.1%, 14.7%, and 30.8%, respectively. These results demonstrate that deploying PCIM consistently across the feature-interaction stages facilitates effective multiscale class-discriminative modeling while improving computational efficiency.

PCIM yields limited gains when applied to a single neck component, with its benefits becoming pronounced only when extended across the neck’s multiscale feature interaction pathways. The complete PCIM configuration establishes unified class-prototype guidance and confusion-aware interaction across scales, strengthening discrimination among similar lesion categories while reducing model complexity and improving the accuracy–efficiency trade-off.

### 4.3. Comparisons on Different Datasets

#### 4.3.1. Results on the HS-CMU Dataset

Table 7 compares the detection performance of ADD-Net with that of several representative object detectors on the HS-CMU dataset, including models from the YOLO family, RT-DETR, and Mamba YOLO-T. Table 8 further compares the model complexity and inference efficiency of ADD-Net with those of the Mamba YOLO-T baseline. All models were evaluated under the same experimental settings to ensure a fair comparison.

As shown in Table 7, in the single-run experiment conducted using a random seed of 0, ADD-Net achieves the highest mAP@50 and mAP@50:95 among all compared methods, reaching 93.5% and 63.3%, respectively. Although RT-DETR obtains the highest recall, ADD-Net is only 0.9 percentage points lower while outperforming it by 2.1 and 3.0 percentage points in mAP@50 and mAP@50:95, respectively. Furthermore, Table 8 shows that ADD-Net reduces the parameter count, FLOPs, and inference latency of the baseline from 7.5 M, 14.8 G, and 2.7 ms to 6.3 M, 12.8 G, and 1.8 ms, corresponding to reductions of approximately 16.0%, 13.5%, and 33.3%, respectively. These results demonstrate the potential of ADD-Net for hysteroscopic lesion detection.

To examine whether the performance gains were sensitive to random initialization, we additionally conducted three independent training runs using random seeds of 0, 21, and 42. Table 9 reports the mean and standard deviation across these runs. Across the three runs, ADD-Net achieves higher mean recall, mAP@50, and mAP@50:95 than Mamba YOLO-T. The corresponding improvements are 2.2, 0.8, and 0.3 percentage points, respectively. These results indicate that ADD-Net maintains reasonably consistent overall detection performance across different random seeds.

ADD-Net improves multiclass hysteroscopic lesion detection while reducing model complexity and inference latency. These advantages arise from the adaptive feature modulation of HAFM, the class-discriminative enhancement of PCIM, and the lesion presence-aware classification calibration of LPC-Head. Together, these components enable a favorable balance between detection accuracy and computational efficiency.

#### 4.3.2. Cross-Dataset Applicability on the Kvasir-Capsule Dataset

To further assess the applicability of ADD-Net across datasets, we independently trained and evaluated ADD-Net and the comparison methods on the Kvasir-Capsule dataset. This dataset differs from HS-CMU in both lesion categories and imaging characteristics, providing a complementary benchmark for evaluating the model in a distinct endoscopic imaging scenario.

As shown in Table 10, ADD-Net achieves a recall of 96.5%, an mAP@50 of 98.5%, and an mAP@50:95 of 72.6%, representing the best results among all compared methods for these three metrics. Compared with the Mamba YOLO-T baseline, ADD-Net improves recall, mAP@50, and mAP@50:95 by 0.3, 0.3, and 0.5 percentage points, respectively. Although YOLO11n achieves higher precision, its mAP@50 and mAP@50:95 are 0.8 and 4.2 percentage points lower than those of ADD-Net. These results indicate that ADD-Net delivers stronger overall detection performance across different IoU thresholds.

Together with the results on HS-CMU, these findings demonstrate that ADD-Net maintains strong detection performance across endoscopic datasets with distinct lesion categories and imaging characteristics. The adaptive feature modulation of HAFM, class-discriminative enhancement of PCIM, and lesion presence-aware classification calibration of LPC-Head work synergistically to improve adaptability across different endoscopic imaging scenarios.

### 4.4. Class-Wise Detection Results on Different Datasets

#### 4.4.1. Class-Wise Results on the HS-CMU Dataset

To further analyze the detection performance of the proposed method across fine-grained lesion categories, we evaluated the average precision (AP) for each class in the HS-CMU dataset. Table 11 compares the class-wise results of Mamba YOLO-T and ADD-Net.

ADD-Net improves detection performance for most lesion categories. The most substantial improvement is observed for the high-risk endometrial cancer (EC) category, for which AP@50 increases from 88.7% to 95.4%. This result indicates that ADD-Net more effectively captures clinically important discriminative features. Performance also improves to varying degrees for endometrial polyp (EP), endometrial polypoid hyperplasia (EPH), and intrauterine foreign body (IFB), indicating that the model can better accommodate variations in lesion morphology and structure. Nevertheless, these improvements are not uniform across all categories. AP@50 decreases by 2.8 percentage points for endometrial hyperplasia without atypia (EH), from 87.3% to 84.5%, and by 2.0 percentage points for atypical endometrial hyperplasia (AHE), from 95.6% to 93.6%. EH and AHE share highly similar color, texture, and morphological characteristics with other endometrial abnormalities, particularly EPH and EC, and their differences may depend on subtle visual cues that are difficult to capture in hysteroscopic images. Because PCIM represents each category using a single learnable prototype, this representation may not fully capture the intraclass appearance variability of these categories. When their feature distributions overlap substantially, prototype interaction may not completely separate these categories in the feature space. Therefore, although ADD-Net improves overall class discrimination and substantially benefits EC detection, the decreases for EH and AHE suggest a remaining class-specific limitation.

ADD-Net increases mAP@50 from 92.6% to 93.5%, demonstrating improved overall detection performance. The ablation and class-wise results further support the effectiveness of the adaptive feature modulation and class-discrimination enhancement modules in fine-grained lesion representation.

#### 4.4.2. Class-Wise Results on the Kvasir-Capsule Dataset

Table 12 compares the class-wise detection performance of Mamba YOLO-T and ADD-Net on Kvasir-Capsule. ADD-Net improves performance across most categories, with the largest AP@50 gain observed for ERO (mucosal erosion), from 94.2% to 96.8%, supporting its effectiveness in detecting lesions with subtle textural cues. ADD-Net also yields modest gains for ANG (angiectasia), FBL (fresh blood), and FOB (foreign body), indicating improved detection across lesions with diverse visual appearances. Performance on ULC and POL remains unchanged, suggesting that feature learning for these categories may be near saturation. The slight decline for LYM may stem from relatively simple visual patterns and weak interclass separability, a local fluctuation that does not alter the overall performance trend.

ADD-Net achieves an mAP@50 of 98.5% and an mAP@50:95 of 72.6% on the Kvasir-Capsule dataset, further demonstrating its robust and broadly consistent performance across fine-grained lesion categories.

### 4.5. Visualization Analysis

To further evaluate the effectiveness of ADD-Net in feature adaptation, class discrimination, and detection performance, we conducted visualization analyses of backbone feature responses, confusion matrices, feature distributions, and qualitative detection results.

As shown by the backbone feature visualizations in Figure 4, introducing HAFM produces stronger responses in lesion-relevant regions while suppressing background interference. Across the $C_{3}$, $C_{4}$, and $C_{5}$ levels, HAFM-modulated features more clearly delineate lesion regions and boundaries, indicating greater backbone adaptability to local texture, structural context, and illumination variations and improved representation quality under complex hysteroscopic conditions.

Figure 5 shows that ADD-Net predictions are more concentrated along the confusion-matrix diagonal, indicating higher recognition accuracy for most classes. Relative to Mamba YOLO-T, ADD-Net provides more accurate classification of EC, EPH, and EH and reduces lesion background confusion, supporting the role of PCIM in sharpening decision boundaries among similar classes and suppressing errors induced by complex background clutter.

The t-SNE [47] visualization in Figure 6 shows interclass overlap and intraclass dispersion in Mamba YOLO-T features, whereas ADD-Net produces tighter intraclass clusters and clearer interclass separation. These distributions suggest that HAFM and PCIM jointly promote the learning of more discriminative lesion representations, enhancing separation among fine-grained classes.

Figure 7 compares the detection results of Mamba YOLO-T and ADD-Net. Across multiple examples, ADD-Net produces more accurate bounding boxes and higher confidence scores, suggesting improved lesion localization and classification.

## 5. Discussion

### 5.1. Main Findings and Underlying Mechanisms

ADD-Net was developed to address challenging imaging interference and confusion among visually similar lesion categories. The experimental results indicate that its performance gains do not arise merely from increased network capacity, but from the complementary contributions of HAFM, PCIM, and LPC-Head at different stages of the detection framework.

As shown in Table 5, removing any expert from HAFM degrades detection performance to varying degrees, supporting the distinct yet complementary roles of the local-texture, structural-context, and illumination-robustness experts. Figure 4 further shows that HAFM-modulated backbone features produce stronger responses within lesion regions and along their boundaries while suppressing background interference. These findings indicate that HAFM dynamically adapts feature representations to the imaging characteristics of each input and are consistent with previous studies showing that adaptive feature processing improves the robustness of medical image models to variations in input distributions [25,26].

The placement experiments show that the benefits of PCIM do not emerge immediately when only a single interaction module is replaced. Instead, its class-discriminative advantage becomes more pronounced as PCIM progressively covers the multiscale feature-interaction pathways in the neck, as shown in Table 6. This finding suggests that introducing class prototypes at isolated locations is insufficient to establish consistent multiscale discriminative representations. In contrast, unified prototype guidance and confusion-relation modeling facilitate class-semantic interaction across different feature scales. The reduced class confusion in Figure 5, together with the improved intraclass compactness and interclass separation in Figure 6, is consistent with the intended role of PCIM. These observations further support the effectiveness of class prototypes and class-relation modeling in enhancing discriminative representations for medical image analysis [27,28,29].

LPC-Head is not designed to independently enhance upstream features. Instead, it uses class-agnostic lesion presence responses to calibrate class predictions. As shown in Table 4, introducing LPC-Head alone provides only limited performance gains. When combined with HAFM and PCIM, however, it further improves both recall and mAP@50. This result indicates that the effectiveness of LPC-Head depends partly on the quality of the upstream features. HAFM and PCIM first improve feature adaptation and class discrimination, respectively, after which LPC-Head converts these enhanced representations into more accurate detection outputs. The qualitative results in Figure 7 further show that the complete model produces more accurate class predictions.

Across three independent runs, ADD-Net improves aggregate detection performance, increasing the mean mAP@50 from 92.5% to 93.3% and the mean mAP@50:95 from 62.9% to 63.2%. However, the class-wise results in Table 11 provide a more nuanced assessment of the class-discriminative modeling in ADD-Net. Although ADD-Net contributes to the substantial improvement observed for EC, the reduced AP@50 values for EH and AHE indicate that prototype-based confusion modeling does not completely resolve the discrimination of all visually similar categories. A single prototype for each category may be insufficient to represent heterogeneous intraclass appearances, particularly when the interclass differences are subtle. This class-specific trade-off motivates future investigation of multiple prototypes per category and distribution-aware prototype learning to better represent intraclass variability and separate highly overlapping endometrial abnormalities.

### 5.2. Cross-Dataset Applicability and Potential Clinical Utility

HS-CMU and Kvasir-Capsule differ substantially in their lesion categories and imaging characteristics. As shown in Table 7 and Table 10, ADD-Net achieves high mAP values on both datasets, indicating that the proposed architecture is applicable to different endoscopic lesion detection scenarios. However, the model was trained and evaluated separately on each dataset. Therefore, these results primarily demonstrate its applicability across different endoscopic tasks rather than zero-shot cross-dataset transfer without retraining.

The class-wise results further reveal the strengths of the proposed model. As shown in Table 11 and Table 12, ADD-Net improves performance for most lesion categories. Particularly notable gains are observed for endometrial cancer and erosion, indicating an improved ability to represent clinically important lesions and lesions with subtle texture patterns. Beyond detection accuracy, Table 8 shows that ADD-Net requires fewer parameters, lower FLOPs, and shorter inference latency than the baseline model, providing a basis for real-time computer-assisted hysteroscopic lesion detection.

### 5.3. Limitations and Future Work

Despite achieving relatively strong detection performance on two endoscopic datasets, ADD-Net has several limitations. Class confusion may still occur for underrepresented lesion categories, lesions with subtle interclass differences, and severely occluded lesions. Moreover, the current evaluation is retrospective and limited to two datasets with restricted institutional diversity. The lack of independent multicenter and prospective validation limits the current evidence for the clinical generalizability of ADD-Net and represents a major barrier to clinical deployment. The robustness and applicability of ADD-Net across different medical centers, imaging devices, and patient populations therefore require further validation.

The decision to restrict the Kvasir-Capsule experiment to clinically relevant and spatially localizable abnormal findings is consistent with the defined objective of multiclass abnormality detection and localization. However, because images showing normal anatomical structures, normal mucosa, or limited-visibility conditions were not included as target-free negative samples, the current experiment does not fully evaluate false-positive suppression on normal or low-quality frames.

Future work will investigate more efficient and dynamic class-relation modeling to improve discrimination of long-tailed categories and highly similar lesions. We will also explore cross-domain generalization, model compression, and temporal video modeling. In addition, ADD-Net will be evaluated on more comprehensive endoscopic data containing normal anatomical structures, target-free frames, limited-visibility observations, and rare findings. Validation using larger multicenter clinical datasets and prospective studies will further assess the robustness, computational efficiency, and practical applicability of ADD-Net.

## 6. Conclusions

This study presented ADD-Net, an efficient adaptive discriminative network for multiclass hysteroscopic lesion detection, designed to mitigate challenging imaging interference and confusion among visually similar lesion categories. ADD-Net integrates three complementary components into the Mamba YOLO-T framework. HAFM dynamically modulates backbone features using local-texture, structural-context, and illumination-robustness experts. PCIM exploits class prototypes and their confusion relationships to sharpen the decision boundaries between similar lesion categories. LPC-Head explicitly models class-agnostic lesion presence responses and uses them to calibrate class predictions. Together, these components improve detection performance through complementary feature adaptation, class discrimination, and prediction calibration.

On the HS-CMU dataset, ADD-Net achieves mean mAP@50 and mAP@50:95 values of 93.3% and 63.2%, respectively, across three independent runs. Compared with the Mamba YOLO-T baseline, it improves the mean mAP@50 by 0.8 percentage points while reducing the parameter count, FLOPs, and inference latency by approximately 16.0%, 13.5%, and 33.3%, respectively. The ablation experiments and visualization results confirm the effectiveness and complementarity of the proposed components. Overall, ADD-Net achieves a favorable balance between detection accuracy and computational efficiency, while demonstrating the potential to adapt to diverse endoscopic imaging scenarios.

## Figures and Tables

**Figure 1:**
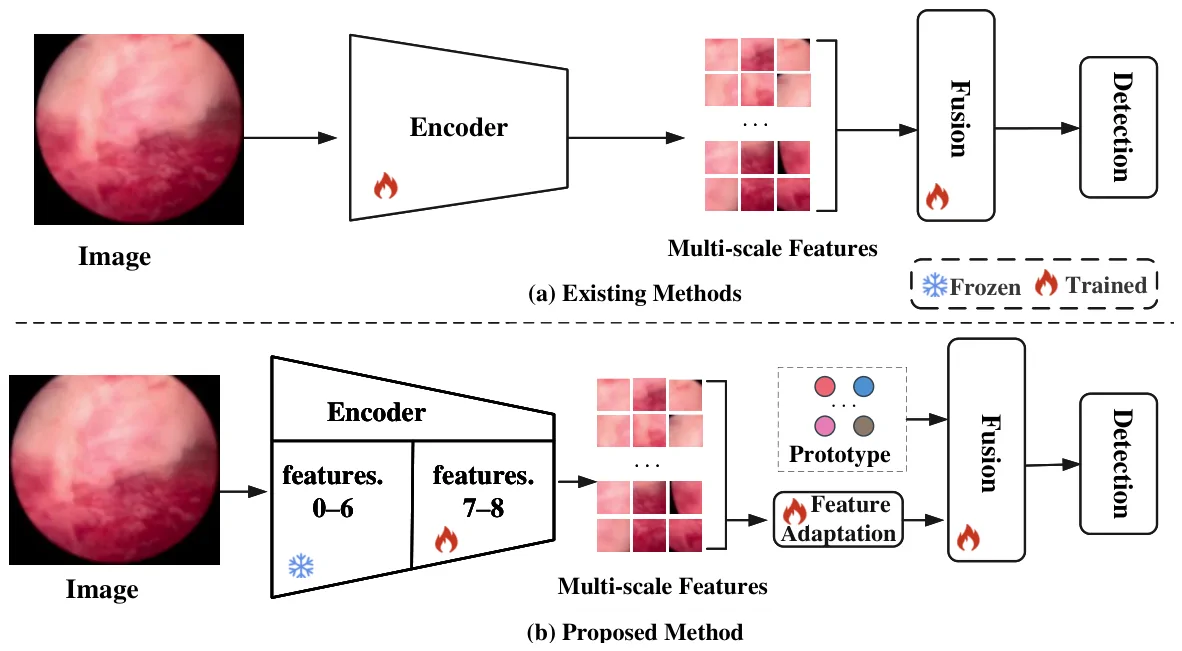
Comparison of feature learning pipelines. (**a**) Existing methods process and directly fuse multiscale encoder features using fixed operations before detection. (**b**) Our proposed ADD-Net introduces adaptive feature modulation and prototype-based confusion modeling to improve feature adaptability and class discrimination.

**Figure 2:**
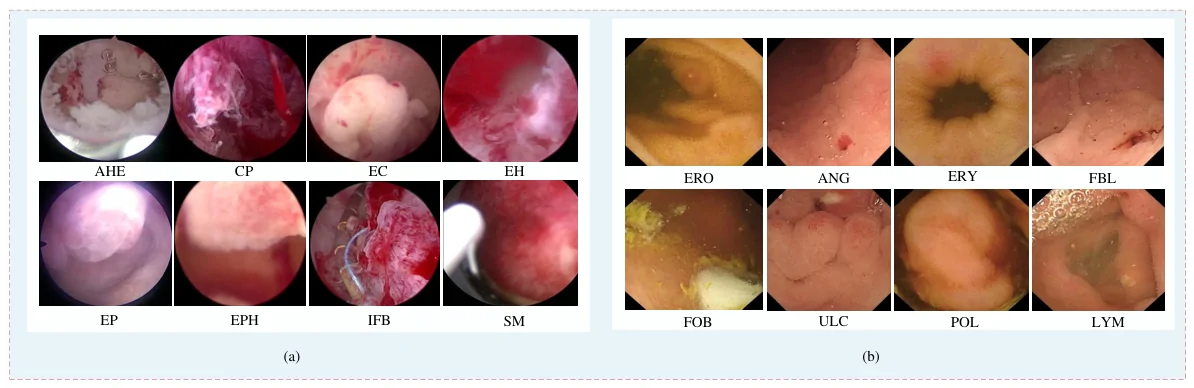
Representative images from the two datasets: (**a**) HS-CMU and (**b**) Kvasir-Capsule.

**Figure 3:**
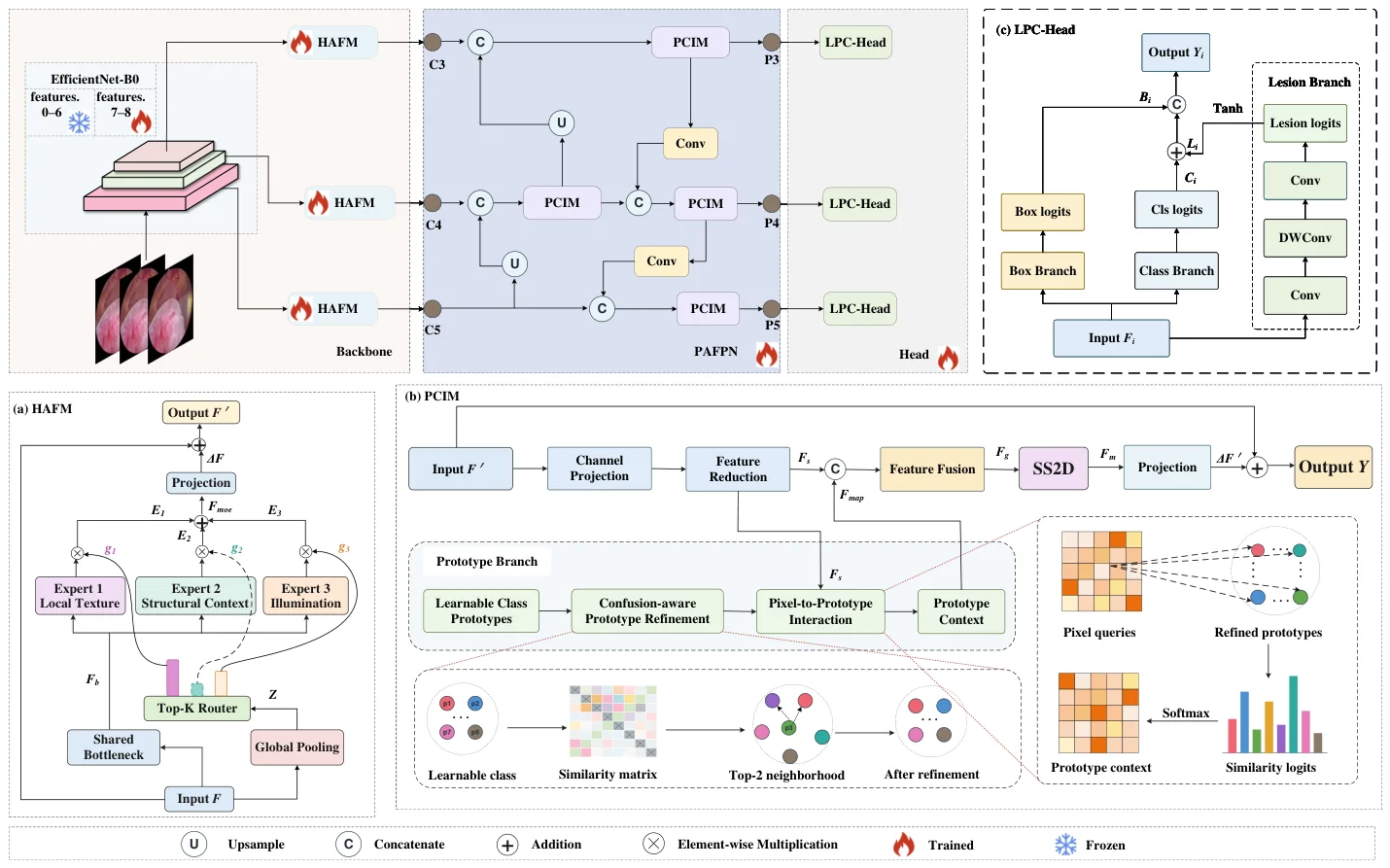
Overall architecture of ADD-Net. EfficientNet-B0 extracts multiscale features for feature fusion and detection at three scales. (**a**) HAFM adaptively modulates the backbone features. (**b**) PCIM refines class prototypes and uses them to enhance feature discrimination. (**c**) LPC-Head generates classification and bounding-box predictions.

**Figure 4:**
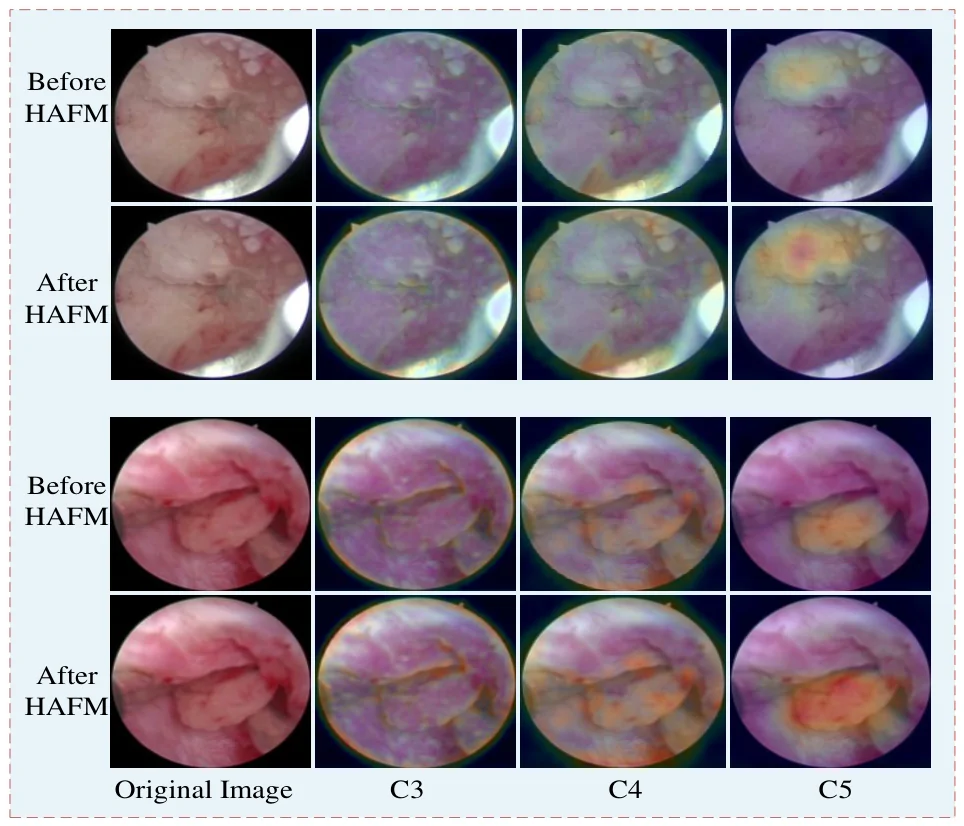
Comparison of multiscale backbone feature responses before and after introducing HAFM at the $C_{3}$, $C_{4}$, and $C_{5}$ stages. In the feature-response visualizations, warmer colors (red) indicate stronger responses.

**Figure 5:**
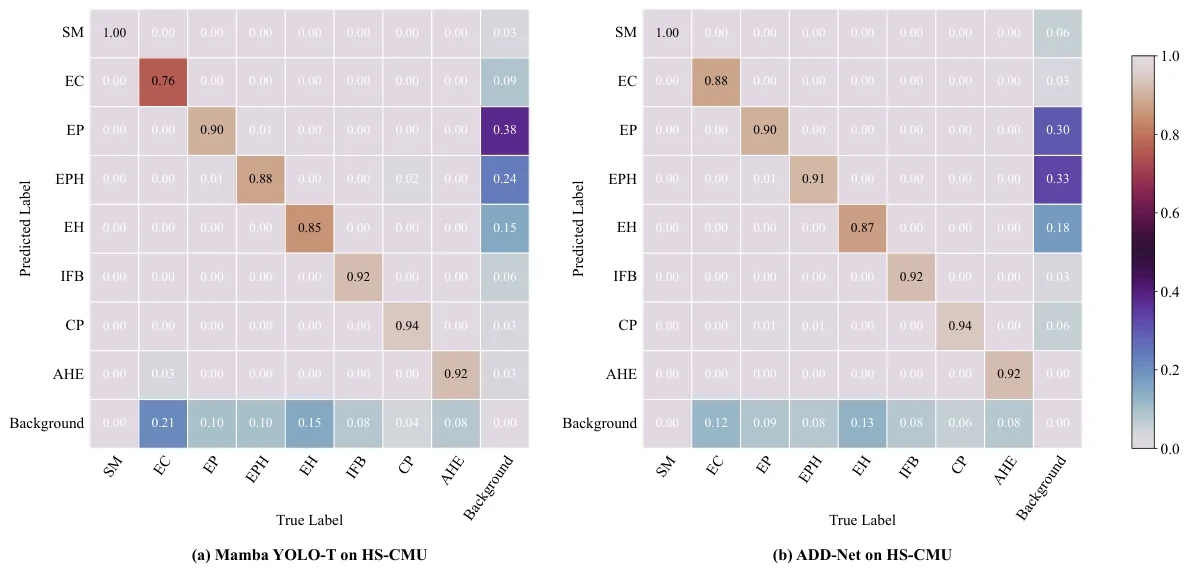
Confusion matrices on the HS-CMU dataset: (**a**) Mamba YOLO-T; (**b**) ADD-Net. Rows represent predicted labels, and columns represent true labels.

**Figure 6:**
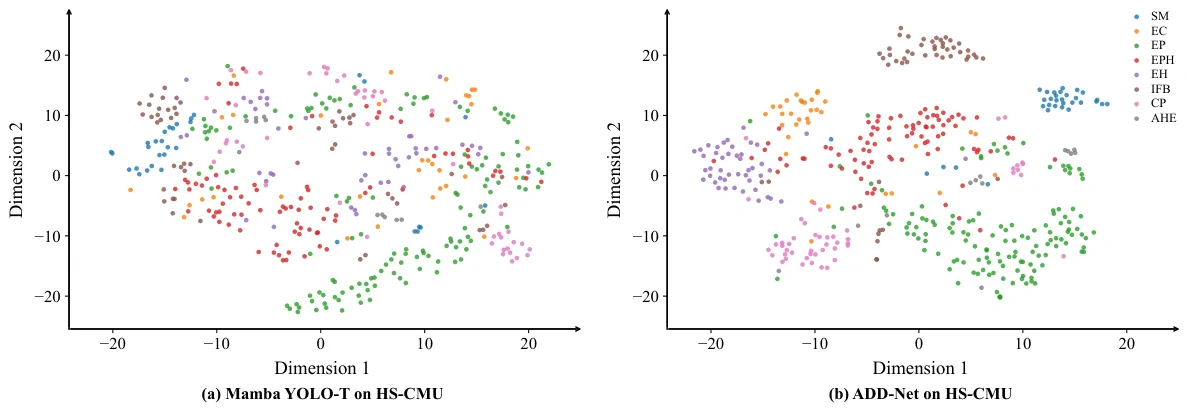
t-SNE visualization of feature distributions on the HS-CMU dataset: (**a**) features learned by Mamba YOLO-T, showing considerable overlap among classes; (**b**) features learned by ADD-Net, forming more compact and distinct class clusters.

**Figure 7:**
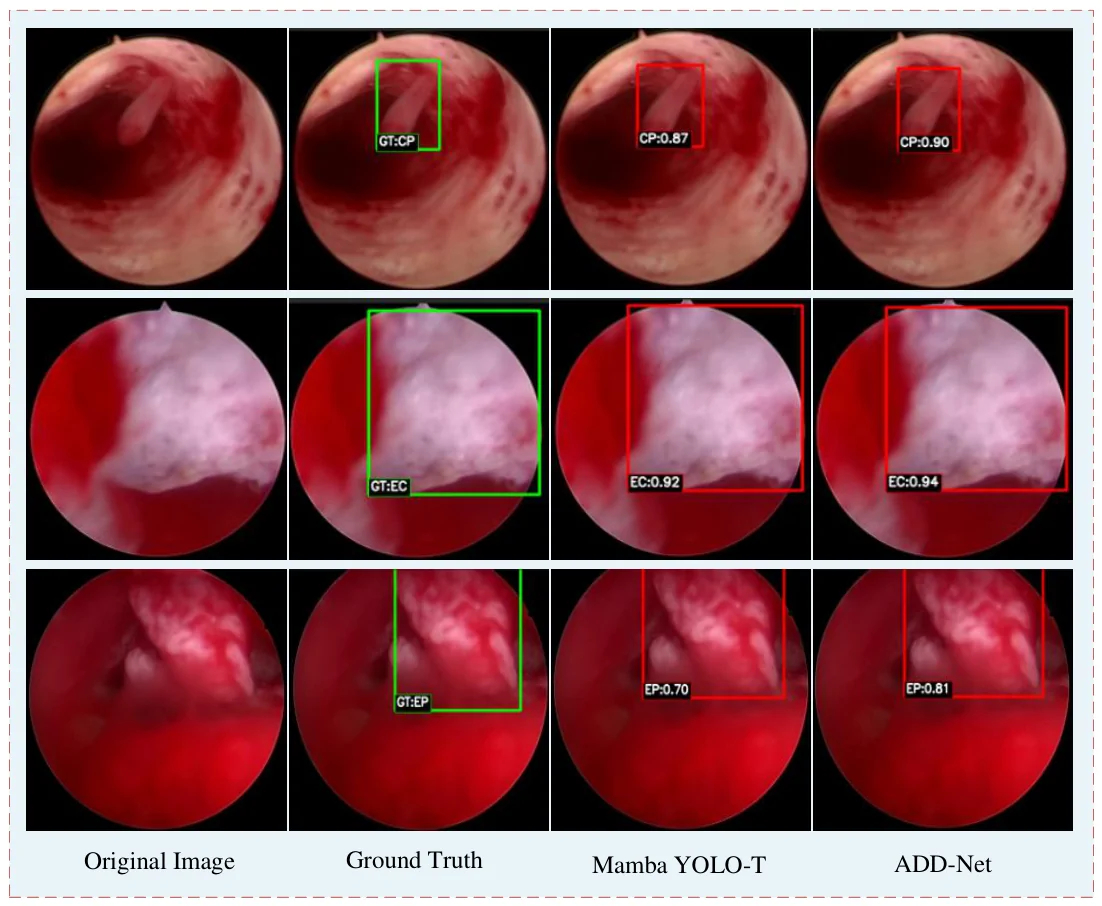
Qualitative comparison of the detection results obtained by Mamba YOLO-T and ADD-Net on the HS-CMU dataset.

**Table 1:** Class-wise distribution of annotated lesion instances in the training, validation, and test sets of the HS-CMU dataset.

Category	Training	Validation	Test	Total
SM	260	32	33	325
EC	293	34	43	370
EP	1138	135	147	1420
EPH	585	86	66	737
EH	313	46	35	394
IFB	388	50	42	480
CP	407	49	50	506
AHE	78	12	10	100
Total	3462	444	426	4332

**Table 2:** Training hyperparameter settings.

Parameter	Value
Input size	640×640
Epochs	120
Batch size	16
Optimizer	SGD
Weight decay	0.0005
Momentum	0.937
Workers	5
Lr0	0.01

**Table 3:** Performance comparison of different backbone networks. Results are based on a single training run using random seed 0.

Backbone	mAP@50 (%)	mAP@50:95 (%)	Params (M)	FLOPs (G)	Latency (ms)
ResNet-18 [35]	91.4	61.8	14.5	37.3	**1.9**
ResNet-34 [35]	92.0	61.7	24.6	67.5	2.2
ResNet-50 [35]	92.5	62.2	27.3	75.9	3.0
DenseNet-121 [36]	92.1	62.5	10.4	54.9	3.3
MobileNet-V2 [37]	91.7	61.1	**5.7**	**12.8**	2.7
RegNetY-800MF [38]	92.0	62.4	9.0	22.2	2.1
ConvNeXt-Tiny [39]	92.4	61.2	31.2	80.5	2.6
EfficientNet-B0 [33]	**92.6**	**62.8**	7.5	14.8	2.7

*Note:* Bold values indicate the best result in each column: the highest mAP values and the lowest parameter count, FLOPs, and latency.

**Table 4:** Ablation results for the proposed components. Results are based on a single training run using random seed 0.

Configuration	Precision (%)	Recall (%)	mAP@50 (%)	Params (M)	Latency (ms)
Mamba YOLO-T	93.0	86.7	92.6	7.5	2.7
+A	88.8	**90.9**	93.0	7.6	2.2
+B	93.0	88.5	93.1	**6.1**	**1.8**
+C	93.3	87.5	92.3	7.5	2.1
+A+B	93.6	88.7	92.9	6.3	2.1
+A+C	94.0	87.0	92.7	7.6	2.6
+B+C	**95.5**	85.7	93.4	6.2	**1.8**
+A+B+C	93.3	89.6	**93.5**	6.3	**1.8**

*Note:* Bold values indicate the best result in each column: the highest precision, recall, and mAP@50, and the lowest parameter count and latency.

**Table 5:** Ablation results for HAFM and its expert branches within ADD-Net. Results are based on a single training run using random seed 0.

Configuration	Precision (%)	Recall (%)	mAP@50 (%)	mAP@50:95 (%)	Params (M)
ADD-Net w/o HAFM	95.5	85.7	93.4	61.6	6.2
ADD-Net w/o LTE	**96.0**	86.7	92.8	62.1	6.3
ADD-Net w/o SCE	94.5	84.5	92.0	63.0	6.3
ADD-Net w/o IRE	90.2	88.7	92.5	62.6	6.3
ADD-Net	93.3	**89.6**	**93.5**	**63.3**	6.3

*Note:* Bold values indicate the best result in each column: the highest precision, recall, mAP@50, and mAP@50:95, and the lowest parameter count.

**Table 6:** Ablation results for different placements of PCIM within ADD-Net. Results are based on a single training run using random seed 0.

Configuration	Recall (%)	mAP@50 (%)	Params (M)	FLOPs (G)	Latency (ms)
ADD-Net w/o PCIM	87.0	92.7	7.6	15.0	2.6
$X_{1}$	86.6	92.5	8.1	15.8	2.3
$X_{1},X_{2}$	87.4	92.1	8.0	14.8	2.1
$X_{1},X_{2},X_{3}$	88.2	93.0	7.1	13.4	1.9
$X_{1},X_{2},X_{3},X_{4}$ (ADD-Net)	**89.6**	**93.5**	**6.3**	**12.8**	**1.8**

*Note:* Bold values indicate the best result in each column: the highest recall and mAP@50, and the lowest parameter count, FLOPs, and latency.

**Table 7:** Comparison with representative detectors on the HS-CMU dataset. Results are based on a single training run using random seed 0.

Method	Precision (%)	Recall (%)	mAP@50 (%)	mAP@50:95 (%)
YOLOv8n [40]	92.9	85.5	89.9	59.0
YOLOv9t [41]	92.1	88.8	92.5	60.4
YOLOv10n [42]	90.8	87.4	90.9	59.2
YOLO11n [43]	90.9	86.1	91.2	58.5
YOLOv12n [44]	91.2	86.1	91.2	59.5
YOLO26n [45]	**93.3**	89.7	92.1	58.8
RT-DETR [46]	90.7	**90.5**	91.4	60.3
Mamba YOLO-T [32]	93.0	86.7	92.6	62.8
ADD-Net (ours)	**93.3**	89.6	**93.5**	**63.3**

*Note:* Bold values indicate the highest value in each metric column.

**Table 8:** Efficiency comparison on the HS-CMU dataset.

Method	Params (M)	FLOPs (G)	Latency (ms)
Mamba YOLO-T	7.5	14.8	2.7
ADD-Net (ours)	**6.3**	**12.8**	**1.8**

*Note:* Bold values indicate the lowest parameter count, FLOPs, and inference latency.

**Table 9:** Performance of Mamba YOLO-T and ADD-Net across three independent training runs on the HS-CMU dataset. Values are reported as mean ± standard deviation.

Method	Precision (%)	Recall (%)	mAP@50 (%)	mAP@50:95 (%)
Mamba YOLO-T	93.6±1.3	87.2±0.9	92.5±0.1	62.9±0.1
ADD-Net (ours)	93.4±0.1	89.4±0.8	93.3±0.3	63.2±0.1

*Note:* Bold entries indicate the higher mean value for each metric.

**Table 10:** Performance comparison of detectors independently trained and evaluated on the Kvasir-Capsule dataset. Results are based on a single training run using random seed 0.

Method	Precision (%)	Recall (%)	mAP@50 (%)	mAP@50:95 (%)
YOLOv8n [40]	98.3	96.1	97.7	68.1
YOLOv9t [41]	98.2	96.0	97.8	68.6
YOLOv10n [42]	96.1	91.6	96.6	67.7
YOLO11n [43]	**98.5**	96.2	97.7	68.4
YOLOv12n [44]	98.0	95.3	97.8	68.8
YOLO26n [45]	98.1	95.4	97.2	68.1
RT-DETR [46]	80.6	83.1	88.4	57.6
Mamba YOLO-T [32]	98.0	96.2	98.2	72.1
ADD-Net (ours)	97.8	**96.5**	**98.5**	**72.6**

*Note:* Bold values indicate the highest value in each metric column.

**Table 11:** Class-wise detection results on the HS-CMU dataset. Results are based on a single training run using random seed 0.

Method	AP@50 (%)								mAP@50 (%)	mAP@50:95 (%)
	SM	EC	EP	EPH	EH	IFB	CP	AHE		
Mamba YOLO-T	**99.5**	88.7	93.6	88.8	**87.3**	89.6	**97.6**	**95.6**	92.6	62.8
ADD-Net (ours)	**99.5**	**95.4**	**94.5**	**90.7**	84.5	**92.4**	97.3	93.6	**93.5**	**63.3**

*Note:* Bold values indicate the highest AP@50 for each lesion category and the highest overall mAP scores.

**Table 12:** Class-wise detection results of models independently trained and evaluated on the Kvasir-Capsule dataset. Results are based on a single training run using random seed 0.

Method	AP@50 (%)								mAP@50 (%)	mAP@50:95 (%)
	ERO	ANG	ERY	FBL	FOB	ULC	POL	LYM		
Mamba YOLO-T	94.2	98.8	**99.5**	99.3	98.8	**95.6**	**99.5**	**99.5**	98.2	72.1
ADD-Net (ours)	**96.8**	**99.2**	**99.5**	**99.4**	**98.9**	**95.6**	**99.5**	99.0	**98.5**	**72.6**

*Note:* Bold values indicate the highest AP@50 for each lesion category and the highest overall mAP scores.

## Data Availability

The original HS-CMU dataset [30] is publicly available at https://openxlab.org.cn/datasets/jiyuanmedical/HS-CMU/ (accessed on 17 September 2026). The original Kvasir-Capsule dataset [31] is publicly available at https://datasets.simula.no/kvasir-capsule/ (accessed on 17 September 2026). The corrected data used in this study are available from the corresponding author upon reasonable request. The source code for the three core modules of ADD-Net is available at https://github.com/Chenzhiwu-ops/ADD-Net (accessed on 17 September 2026).

## References

[1] Clark C., Cazzolla A., Colonna G., Loizzi V., Cormio G., Lopez S. (2025). Hysteroscopy vs. vabra in endometrial cancer diagnosis: A systematic review of the literature. Cancers.

[2] Neofytou M.S., Tanos V., Pattichis M.S., Kyriacou E.C., Pattichis C.S., Schizas C.N. (2008). Color multiscale texture classification of hysteroscopy images of the endometrium. Proceedings of the 2008 30th Annual International Conference of the IEEE Engineering in Medicine and Biology Society.

[3] Neofytou M., Tanos V., Constantinou I., Kyriacou E., Pattichis M., Pattichis C. (2015). Computer-Aided Diagnosis in Hysteroscopic Imaging. IEEE J. Biomed. Health Inform..

[4] Vlachokosta A.A., Asvestas P.A., Gkrozou F., Lavasidis L., Matsopoulos G.K., Paschopoulos M. (2013). Classification of hysteroscopical images using texture and vessel descriptors. Med. Biol. Eng. Comput..

[5] Cortes C., Vapnik V. (1995). Support-vector networks. Mach. Learn..

[6] Cover T., Hart P. (1967). Nearest neighbor pattern classification. IEEE Trans. Inf. Theory.

[7] Takahashi Y., Sone K., Noda K., Yoshida K., Toyohara Y., Kato K., Inoue F., Kukita A., Taguchi A., Nishida H. (2021). Automated system for diagnosing endometrial cancer by adopting deep-learning technology in hysteroscopy. PLoS ONE.

[8] Mascarenhas M., Peixoto C., Freire R., Cavaco Gomes J., Cardoso P., Castro I., Martins M., Mendes F., Mota J., Almeida M.J. (2025). Artificial intelligence and hysteroscopy: A multicentric study on automated classification of pleomorphic lesions. Cancers.

[9] Wang W., Cai Y., Guo Z., Zhao A., Ma W., Wang W., Wang S., Zhu X., Du X., Shen W. (2025). A deep learning-based computer-aided diagnosis system for detecting atypical endometrial hyperplasia and endometrial cancer through hysteroscopy. iScience.

[10] Li B., Chen H., Duan H. (2025). Visualized hysteroscopic artificial intelligence fertility assessment system for endometrial injury: An image-deep-learning study. Ann. Med..

[11] Watrowski R., Di Spiezio Sardo A., Török P., Rosati A., Kostov S., Alkatout I., Vitale S.G. (2026). Artificial Intelligence, Deep Learning, and Computer Vision in Hysteroscopy: A Systematic Review. Diagnostics.

[12] Zhao A., Zhang J., Wang S., Wang Y., Zhu X., Shen W., Wang W. (2021). Detection of hysteroscopic hysteromyoma in Real-Time based on deep learning. J. Phys. Conf. Ser..

[13] Zhao A., Du X., Yuan S., Shen W., Zhu X., Wang W. (2023). Automated detection of endometrial polyps from hysteroscopic videos using deep learning. Diagnostics.

[14] Rostami G., Hosseini Berneti S.H., Habibzadeh N., Bazir M. (2025). From colon to uterus: Potential of YOLOv7 for Real-Time polyp detection in hysteroscopy. Infosci. Trends.

[15] Selçuk B., Tekeli A., Serif T., Baylan I.Y.Y., Attar E. (2025). Deep Learning-Based Segmentation of Hysteroscopic Images for Early Detection of Endometrial Cancer. Proceedings of the 2025 Fourteenth International Conference on Image Processing, Theory, Tools & Applications (IPTA).

[16] Chen X. (2025). HD-YOLO: Real-time detection of benign and malignant endometrial lesions using YOLO11. Signal Image Video Process..

[17] Zhang J., Peng X.L. (2026). YOLOv11-LC: Accurate detection of benign and malignant endometrial lesions using LCA-enhanced YOLOv11 with CMA-C3k2. Signal Image Video Process..

[18] Chen X., Wang G., Cao J., Lu Y. (2026). HL-Det: A multi-scale adaptive network integrating spatial-frequency features for endometrial lesion detection. Biomed. Signal Process. Control.

[19] Neha F., Shukla D.K. (2026). Radiomics in Medical Imaging: Methods, Applications, and Challenges. J. Imaging.

[20] Zhang Y., Wang Z., Zhang J., Wang C., Wang Y., Chen H., Shan L., Huo J., Gu J., Ma X. (2021). Deep learning model for classifying endometrial lesions. J. Transl. Med..

[21] Raimondo D., Raffone A., Salucci P., Raimondo I., Capobianco G., Galatolo F.A., Cimino M.G.C.A., Travaglino A., Maletta M., Ferla S. (2024). Detection and classification of hysteroscopic images using deep learning. Cancers.

[22] Taher M.R.H., Haghighi F., Gotway M.B., Liang J. (2025). Large-scale benchmarking and boosting transfer learning for medical image analysis. Med. Image Anal..

[23] Valverde J.M. (2026). Fine-tuning ImageNet-pretrained models in medical image classification: Reassessing the impact of different factors. Pattern Recognit. Lett..

[24] Okazaki S., Mine Y., Yoshimi Y., Iwamoto Y., Ito S., Peng T.Y., Nishimura T., Suehiro T., Koizumi Y., Nomura R. (2025). RadImageNet and ImageNet as datasets for transfer learning in the assessment of dental radiographs: A comparative study. J. Imaging Inform. Med..

[25] Li X., Lan L., Lahza H., Yang S., Wang S., Yang W., Liu H., Zhang Y. (2024). EAFP-Med: An efficient adaptive feature processing module based on prompts for medical image detection. Expert Syst. Appl..

[26] Cai Z., Chen Y., Wang J., He X., Pei Z., Lei X., Lu C. (2025). DAFNet: A novel Dynamic Adaptive Fusion Network for medical image classification. Inf. Fusion.

[27] Zhang Y., Li H., Gao Y., Duan H., Huang Y., Zheng Y. (2024). Prototype correlation matching and class-relation reasoning for few-shot medical image segmentation. IEEE Trans. Med. Imaging.

[28] Song Y., Xu C., Wang B., Du X., Chen J., Zhang Y., Li S. (2025). Interactive prototype learning and self-learning for few-shot medical image segmentation. Artif. Intell. Med..

[29] Wang C., Liu F., Chen Y., Frazer H., Carneiro G. (2025). Cross-and intra-image prototypical learning for multi-label disease diagnosis and interpretation. IEEE Trans. Med. Imaging.

[30] Han R., Xie Y., You K., Cao L., Li H. (2024). The HS-CMU dataset for diagnosing benign and malignant diseases through hysteroscopy. arXiv.

[31] Smedsrud P.H., Thambawita V., Hicks S.A., Gjestang H., Nedrejord O.O., Næss E., Borgli H., Jha D., Berstad T.J.D., Eskeland S.L. (2021). Kvasir-Capsule, a video capsule endoscopy dataset. Sci. Data.

[32] Wang Z., Li C., Xu H., Zhu X., Li H. (2025). Mamba yolo: A simple baseline for object detection with state space model. Proc. AAAI Conf. Artif. Intell..

[33] Tan M., Le Q. (2019). Efficientnet: Rethinking model scaling for convolutional neural networks. Proceedings of the International Conference on Machine Learning.

[34] Liu Y., Tian Y., Zhao Y., Yu H., Xie L., Wang Y., Ye Q., Jiao J., Liu Y. (2024). Vmamba: Visual state space model. Adv. Neural Inf. Process. Syst..

[35] He K., Zhang X., Ren S., Sun J. (2016). Deep residual learning for image recognition. Proceedings of the IEEE Conference on Computer Vision and Pattern Recognition.

[36] Huang G., Liu Z., Van Der Maaten L., Weinberger K.Q. (2017). Densely connected convolutional networks. Proceedings of the IEEE Conference on Computer Vision and Pattern Recognition.

[37] Sandler M., Howard A., Zhu M., Zhmoginov A., Chen L.C. (2018). Mobilenetv2: Inverted residuals and linear bottlenecks. Proceedings of the IEEE Conference on Computer Vision and Pattern Recognition.

[38] Radosavovic I., Kosaraju R.P., Girshick R., He K., Dollár P. (2020). Designing network design spaces. Proceedings of the IEEE/CVF Conference on Computer Vision and Pattern Recognition.

[39] Liu Z., Mao H., Wu C.Y., Feichtenhofer C., Darrell T., Xie S. (2022). A convnet for the 2020s. Proceedings of the IEEE/CVF Conference on Computer Vision and Pattern Recognition.

[40] Jocher G., Chaurasia A., Qiu J. Ultralytics YOLOv8, Version 8.0.0, 2023. https://github.com/ultralytics/ultralytics.

[41] Wang C.Y., Yeh I.H., Mark Liao H.Y. (2025). Yolov9: Learning what you want to learn using programmable gradient information. Proceedings of the European Conference on Computer Vision.

[42] Wang A., Chen H., Liu L., Chen K., Lin Z., Han J., Ding G. (2024). Yolov10: Real-time end-to-end object detection. Adv. Neural Inf. Process. Syst..

[43] Jocher G., Qiu J. Ultralytics YOLO11, Version 11.0.0, 2024. https://github.com/ultralytics/ultralytics.

[44] Tian Y., Ye Q., Doermann D. (2026). Yolov12: Attention-centric real-time object detectors. Adv. Neural Inf. Process. Syst..

[45] Jocher G., Qiu J., Liu M., Lyu S., Akyon F.C., Kalfaoglu M.E. (2026). Ultralytics YOLO26: Unified Real-Time End-to-End Vision Models. arXiv.

[46] Zhao Y., Lv W., Xu S., Wei J., Wang G., Dang Q., Liu Y., Chen J. (2024). DETRs beat YOLOs on real-time object detection. Proceedings of the IEEE/CVF Conference on Computer Vision and Pattern Recognition.

[47] Van der Maaten L., Hinton G. (2008). Visualizing data using t-SNE. J. Mach. Learn. Res..

